# Preoperative inflammation-based biomarkers and postoperative pulmonary complications after lung resection for lung cancer: a systematic review with selective exploratory quantitative synthesis

**DOI:** 10.3389/fsurg.2026.1853516

**Published:** 2026-07-30

**Authors:** Xiaoqing Zhu, Chuan Long, Xiaojiao Zhu, Suwei Xu, Wei Shao

**Affiliations:** 1 Department of Endocrinology, The First People’s Hospital of Jiande, Jiande, China; 2 Department of Thoracic Surgery, The First People’s Hospital of Jiande, Jiande, China; 3 Department of Oncology, The First People’s Hospital of Jiande, Jiande, China

**Keywords:** inflammation-based biomarkers, lung cancer, lung resection, postoperative pulmonary complications, systematic review

## Abstract

**Background:**

Postoperative pulmonary complications (PPCs) remain a source of morbidity after lung resection for lung cancer. Several preoperative inflammation-based biomarkers have been investigated as potential risk indicators, but the literature remains methodologically heterogeneous across biomarker types, analytic scales, and outcome definitions.

**Methods:**

Following PRISMA 2020 guidelines, we searched PubMed, Web of Science, Scopus, Embase, and the Cochrane Library from inception to March 20, 2026. We included original studies evaluating preoperative inflammation-based biomarkers in patients undergoing lung resection for lung cancer if PPCs or extractable pulmonary complication outcomes were reported. PPCs were prespecified as the primary outcome framework, and postoperative pneumonia (POP) was analyzed as a major subtype. Because of substantial heterogeneity across biomarkers, analytical approaches, and outcome definitions, the evidence was synthesized at the study level, with selective exploratory quantitative syntheses performed only when clinical comparability was acceptable.

**Results:**

Eight studies were included, comprising seven retrospective cohorts and one prospective cohort. Quantitative synthesis was exploratory because biomarker selection, analytic scale, and pulmonary outcome definitions varied substantially across studies. Pooled analyses based on extractable effect estimates were generally unstable and highly heterogeneous; for example, the biomarker-specific NLR analysis was not significant (OR 1.53, 95% CI 0.79–2.99; I^2^ = 96%), and the pooled all-estimates analysis remained highly heterogeneous (OR 1.04, 95% CI 1.00–1.08; I^2^ = 92%). Restriction to adjusted estimates did not materially reduce this inconsistency (OR 1.01, 95% CI 0.97–1.04; I^2^ = 94%). By contrast, an exploratory dichotomous synthesis of cutoff-defined biomarker categories suggested higher pulmonary risk in patients with elevated preoperative biomarker levels (OR 1.81, 95% CI 1.33–2.47; I^2^ = 0%), although this result was based on crude data across different biomarkers and should be interpreted cautiously. At the study level, CRP, SII, and NLR showed the most consistent adverse associations.

**Conclusions:**

Selected preoperative inflammation-based biomarkers, particularly CRP, SII, and NLR, may be associated with postoperative pulmonary morbidity after lung cancer resection. However, the literature is dominated by retrospective studies and marked methodological heterogeneity, precluding a single robust pooled inference. Prospective biomarker-specific studies with standardized pulmonary outcome definitions are needed before these markers can be considered for perioperative risk stratification.

**Systematic Review Registration:**

https://www.crd.york.ac.uk/PROSPERO/view/CRD420261356969, PROSPERO CRD420261356969.

## Introduction

Lung cancer remains a major global health burden and continues to account for the greatest number of cancer-related deaths worldwide (1). For patients with resectable disease, lung resection remains the cornerstone of curative-intent treatment; however, postoperative pulmonary complications (PPCs) continue to be a major source of morbidity after surgery (2). PPCs such as pneumonia, atelectasis, respiratory failure, and prolonged air leak can delay recovery, increase healthcare utilization, and adversely affect early postoperative outcomes (2, 3). Despite advances in perioperative care and enhanced recovery pathways, reliable preoperative identification of patients at increased pulmonary risk remains an unresolved challenge (3, 4).

Against this background, preoperative inflammation-based biomarkers have attracted increasing attention because they are inexpensive, routinely available, and biologically plausible. Systemic inflammation, immune dysregulation, and impaired nutritional reserve may all contribute to pulmonary infection, exaggerated inflammatory injury, and delayed respiratory recovery after lung resection. CRP has been associated with postoperative pulmonary risk in thoracic surgical cohorts (5, 6), whereas blood cell–derived indices such as NLR, PLR, SII, and LMR have been investigated in several lung cancer surgical series (7–12). In parallel, albumin- and prealbumin-related measures have also been explored as inflammation-nutrition indicators in relation to postoperative pulmonary outcomes (7, 10).

However, the existing literature remains difficult to interpret. Some studies have reported associations between elevated CRP, SII, NLR, PLR, or LMR and postoperative pulmonary morbidity after lung cancer surgery (5, 6, 8–12), whereas others have shown weaker or less consistent findings (7). The available studies also differ substantially in biomarker selection, analytic scale, effect measure, and endpoint definition. Some investigators evaluated composite PPCs, whereas others focused specifically on postoperative pneumonia (POP) (5, 8, 10–12). Some reported multivariable-adjusted estimates (5, 8, 10–12), whereas others relied on univariable or cutoff-based analyses (6, 9). These differences have limited cross-study comparability and have made it difficult to determine whether preoperative inflammation-based biomarkers provide a reproducible clinical signal across the current evidence base.

To address this evidence gap, we conducted a systematic review with selective exploratory quantitative synthesis to examine the association between preoperative inflammation-based biomarkers and postoperative pulmonary complications after lung resection for lung cancer. PPCs were prespecified as the primary outcome framework, and postoperative pneumonia (POP) was treated as a major subtype within that framework. Our aim was not to force a single summary effect across dissimilar biomarkers, but to identify where the current evidence shows consistent study-level signals, where it remains unstable because of methodological heterogeneity, and which biomarkers may justify further prospective validation.

## Methods

### Protocol and reporting standard

This systematic review with selective exploratory quantitative synthesis was conducted in accordance with the Preferred Reporting Items for Systematic Reviews and Meta-Analyses (PRISMA) 2020 statement (13) and the methodological recommendations of the Cochrane Handbook for Systematic Reviews of Interventions (14). The review protocol was prospectively registered in the International Prospective Register of Systematic Reviews (PROSPERO; registration No. CRD420261356969).

### Search strategy

A comprehensive literature search was independently performed by two reviewers in PubMed, Embase, Web of Science Core Collection, Scopus, and the Cochrane Library from database inception to 20 March 2026. The search strategy combined controlled vocabulary and free-text terms related to lung cancer, lung resection, postoperative pulmonary complications, postoperative pneumonia, and preoperative inflammation-based biomarkers. Search terms were adapted to the indexing system and syntax of each database. In addition, the reference lists of all eligible studies and relevant review articles were manually screened to identify any additional studies. The detailed search strategies for all databases are provided in the Supplementary Material.

### Eligibility criteria

Studies were included if they met the following criteria: (1) adult patients underwent lung resection for lung cancer; (2) at least one preoperative inflammation-based biomarker was evaluated, including inflammatory, immune-inflammatory, or inflammation-nutrition blood-based markers measured before surgery; (3) postoperative pulmonary complications (PPCs) were explicitly reported, or pulmonary complication outcomes could be extracted separately from the full text; and (4) the study provided at least one eligible form of data for synthesis or descriptive analysis, including odds ratios (ORs), risk ratios (RRs), hazard ratios (HRs), raw frequency data, continuous biomarker differences between complication groups, or predictive performance measures such as area under the curve (AUC), cutoff, sensitivity, and specificity. PPCs were prespecified as the primary outcome framework. Postoperative pneumonia (POP) was considered a major subtype within the PPC framework and was analyzed separately when appropriate.

Studies were excluded if they: (1) included non-lung cancer populations or mixed thoracic surgical populations without extractable lung cancer data; (2) involved non-resection procedures only; (3) evaluated only postoperative biomarkers, perioperative biomarker changes, or postoperative biomarker trajectories without preoperative inflammatory marker results; (4) reported only overall postoperative complications, cardiopulmonary complications, or infectious complications without extractable pulmonary complication data; (5) lacked extractable data for the association between preoperative inflammation-based biomarkers and PPCs; or (6) were reviews, systematic reviews, meta-analyses, editorials, letters, comments, case reports, conference abstracts, conference papers, posters, oral presentations, animal studies, or basic science studies. When overlapping cohorts were suspected, the most informative study with the most complete data and the best fit to the present review question was retained.

### Study selection

Two reviewers independently screened all retrieved records in two stages. After removal of duplicates, titles and abstracts were screened for potential relevance. Full texts of potentially eligible studies were then assessed against the predefined inclusion and exclusion criteria. Disagreements were resolved through discussion, with consultation of a third reviewer when necessary.

### Data extraction

Data were independently extracted by two reviewers using a standardized form. The following information was collected: first author, publication year, country, study design, patient population, sample size, surgical procedure, biomarker type, biomarker definition or cutoff, timing of biomarker measurement, outcome definition, and reported association measures. When available, multivariable-adjusted effect estimates were extracted preferentially over univariable estimates. If adjusted estimates were unavailable, univariable estimates or raw frequency data were recorded. For studies reporting more than one eligible biomarker, each eligible estimate was recorded separately in advance, while interpretation remained anchored to the original source study.

Because this review addressed prognostic or risk-factor associations rather than intervention effects, several comparison formats were considered eligible, including PPC vs. non-PPC groups, high vs. low biomarker groups, and continuous biomarker associations with PPC risk according to the original study design. Studies reporting clinically informative but non-independent estimates, such as multiple biomarkers from the same study or multiple estimates sharing a common patient cohort, were retained for descriptive synthesis and exploratory analyses but were interpreted cautiously.

### Quality assessment

The methodological quality of included non-randomized studies was independently assessed by two reviewers using the Newcastle–Ottawa Scale (NOS), which evaluates study quality across the domains of selection, comparability, and outcome, with scores ranging from 0 to 9. Discrepancies were resolved by discussion with a third reviewer when necessary.

### Outcome definitions

The primary outcome of this review was postoperative pulmonary complications (PPCs) after lung resection for lung cancer. PPCs were defined according to the original study definitions and could include composite pulmonary complication outcomes or clearly pulmonary postoperative events such as postoperative pneumonia, atelectasis, respiratory failure, prolonged air leak, pleural effusion, empyema, bronchopleural fistula, prolonged mechanical ventilation due to pulmonary causes, reintubation for pulmonary causes, or acute respiratory distress syndrome. POP was treated as a major PPC subtype and was synthesized separately when sufficient comparable data were available. Other individually reported pulmonary outcomes were summarized descriptively when quantitative pooling was not appropriate.

### Statistical analysis

Meta-analyses were performed using Review Manager (RevMan) version 5.4.1. Because substantial clinical and methodological heterogeneity was anticipated across biomarkers, analytical scales, and pulmonary outcome definitions, quantitative synthesis was considered exploratory and was undertaken selectively only when clinical comparability was judged to be acceptable. Odds ratios (ORs) with 95% confidence intervals (CIs) were used for effect-estimate-based analyses. For generic inverse-variance analyses, ORs were transformed into log(OR) values with corresponding standard errors. Statistical heterogeneity was assessed using Cochran's *Q* test and the I^2^ statistic. A fixed-effect model was used when heterogeneity was low, whereas a random-effects model was applied when substantial heterogeneity was present. A two-sided *P*-value < 0.05 was considered statistically significant.

Different data structures were analyzed separately. Effect-estimate-based analyses derived from regression models were not combined with crude dichotomous 2 × 2 data or continuous-difference data within the same pooled estimate. Likewise, different biomarkers were not prespecified to contribute to one unified primary pooled effect. Biomarker-specific synthesis was preferred whenever feasible; when only a small number of studies were available for a given biomarker, findings were summarized descriptively or interpreted as exploratory.

Where sufficient clinical similarity was judged to be present, limited exploratory quantitative syntheses were undertaken to examine whether broadly consistent directional signals could be observed. These analyses were considered hypothesis-generating only and were not intended to provide a definitive pooled effect across biomarkers. When a single study reported more than one eligible biomarker estimate, these estimates were considered separately for descriptive and exploratory purposes; however, they were not interpreted as statistically independent confirmations of the same signal. Accordingly, all such quantitative findings were treated as secondary and hypothesis-generating. In addition, an exploratory dichotomous meta-analysis was performed for studies with extractable 2 × 2 data for cutoff-based biomarker categories, while avoiding double-counting of overlapping estimates from the same cohort. Formal assessment of publication bias was not performed because fewer than 10 studies were available for any pooled analysis.

## Results

### Study selection and characteristics

**Figure 1:**
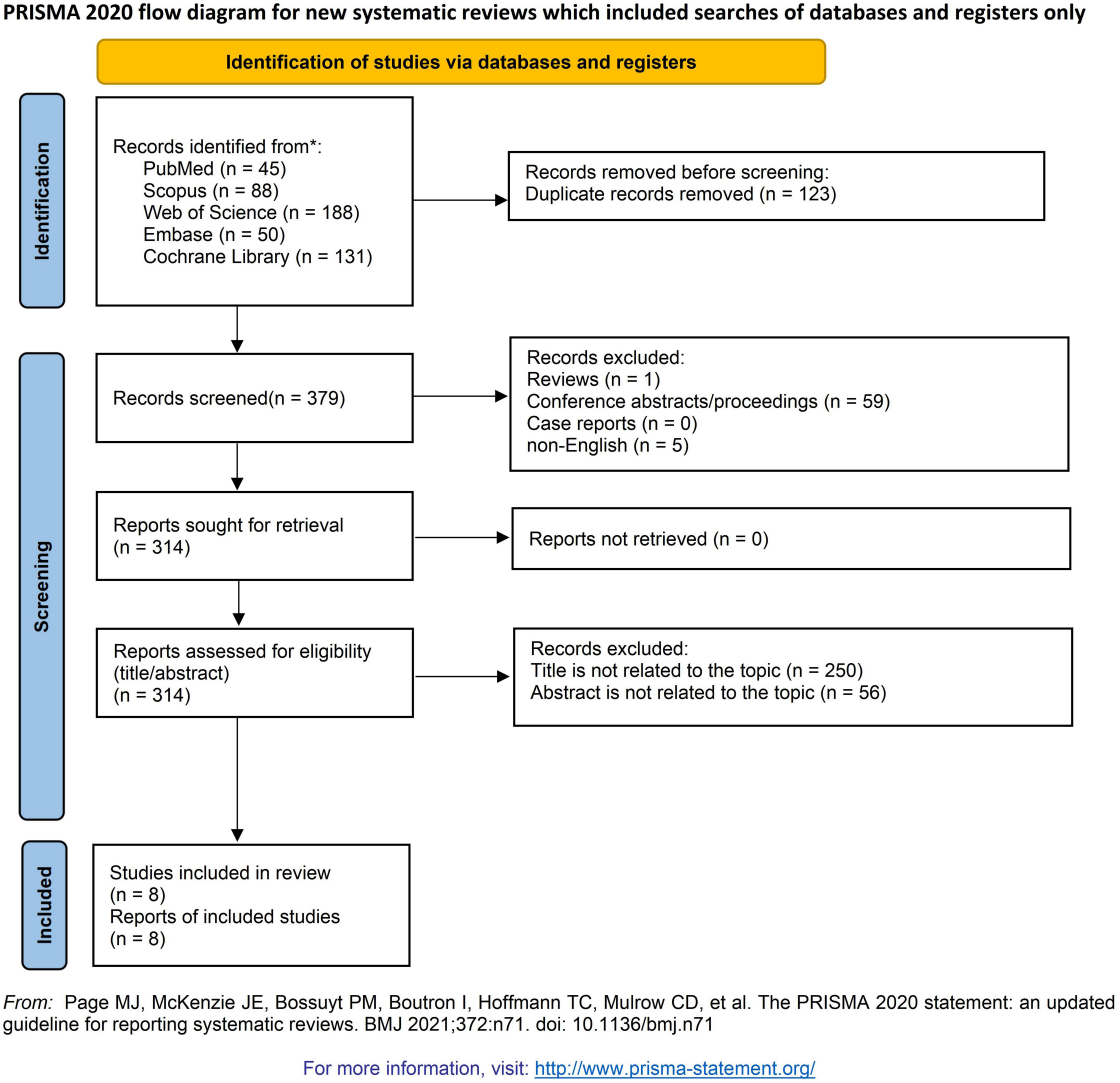
The PRISMA flow diagram displays the details of the selection process. *From: Page, M.J., McKenzie, J.E., Bossuyt, P.M., Boutron, I., Hofmann, T.C., Mulrow, C.D., Shamseer, L., Tetzlaf, J.M., Akl, E.A., Brennan, S.E., et al. (2021). The PRISMA 2020 statement: an updated guideline for reporting systematic reviews. Syst Rev. Mar 29;10(1):89. doi: 10.1186/s13643-021-01626-4. PMID: 33781348; PMCID: PMC8008539.

From database inception to 20 March 2026, the systematic search identified 502 records. After removal of 123 duplicates, 379 records were screened by title and abstract, and 314 full-text reports were assessed for eligibility. Ultimately, eight studies met the inclusion criteria and were included in the systematic review (Figure 1) (5–12). Seven studies contributed data to at least one exploratory quantitative synthesis, whereas Mizuno et al. (6) was retained for qualitative synthesis only because it evaluated postoperative acute exacerbation of idiopathic pulmonary fibrosis rather than standard PPCs or POP in the general lung cancer surgery population.

The included evidence base was dominated by retrospective cohort studies, comprising seven retrospective cohorts (5–8, 10–12) and one prospective cohort (9). Most studies were conducted in Asia, including China, Taiwan, and Japan, whereas Baar et al. (5) reported a multicenter retrospective cohort from Germany. The biomarkers assessed varied substantially across studies and included CRP (5, 6), NLR (7, 9, 12), PLR (7, 9, 11), SII (8, 10), prealbumin (10), LMR (11), and albumin (7). Pulmonary outcome definitions also differed across studies: some investigators reported composite PPCs, whereas others focused specifically on POP (5, 8, 10–12). Key study characteristics are summarized in Table 1, detailed extracted data are presented in Supplementary Table S1, and study quality assessment using the Newcastle–Ottawa Scale is shown in Table 2. Overall, the included studies were of moderate to high methodological quality.

**Table 1:** Main characteristics of the studies included in the systematic review and exploratory quantitative synthesis.

Authors	Year	Country	Study design	Population/disease entity	Total (n)	Biomarker(s) assessed	Biomarker measurement timing	Comparator/grouping strategy	Pulmonary outcome definition/main outcome reported	Surgical procedure/approach	Main clinicopathological variables reported	Main extractable result type
Baar et al. (5)	2022	Germany	Multicenter retrospective cohort study	Patients undergoing open thoracotomy lung resection for primary lung cancer	1426	CRP; FEV1	Preoperative assessment	PPC vs. no PPC; multivariable stepwise logistic regression	Composite PPC outcome, including prolonged air leak ≥7 days, postoperative pneumonia, non-invasive ventilation due to respiratory failure, new chest drain, re-intubation/continued ventilation, pleural empyema, and ECMO	Open thoracotomy lung resection (pneumonectomy, lobectomy, bilobectomy, segmentectomy, wedge resection, and bronchoplasty)	Sex; age; BMI; smoking history; ASA class; neoadjuvant therapy; pulmonary infection within 4 weeks before surgery; CRP; hemoglobin; blood gas variables; FEV1; operative and anesthetic variables	Adjusted ORs with 95% CIs; raw frequency data extractable
Chang et al. (7)	2023	Taiwan	Single-center retrospective cohort study	Patients with resectable stage I-IIIA NSCLC undergoing curative-intent lung resection	298	Albumin; NLR; PLR	Within 1 week before surgery	PPC vs. no PPC; multivariable logistic regression using continuous biomarkers	PPCs within 30 days after surgery, including atelectasis, ARDS, emphysema, pneumonia, pleural effusion, pulmonary embolism, prolonged air leak ≥7 days, and re-intubation	Lobectomy, wedge resection, or segmentectomy via VATS or open thoracotomy	Age; sex; smoking history; mFI-5; pathologic stage; BMI; albumin; sarcopenia; surgical approach; resection type; treatment modality	Adjusted ORs for PPCs available, but not directly for the target inflammation-based biomarkers; raw frequency data extractable
Jiang et al. (8)	2023	China	Single-center retrospective cohort study	Patients with NSCLC undergoing pulmonary resection	1486	SII; NLR; PLR	1 day before surgery	POP vs. no POP; continuous SII in multivariable logistic regression; ROC analysis	POP within 30 days after surgery, defined by at least 3 clinical, radiographic, laboratory, or microbiologic criteria	Pulmonary resection for NSCLC via VATS or open thoracotomy	Sex; age; smoking history; diabetes; hypertension; CHD; COPD; BMI; stage; histology; FEV1; DLco; operative time; blood loss; surgical approach	Adjusted OR for continuous SII; AUC and cut-off performance; raw frequency data extractable
Lan et al. (9)	2017	China	Single-center prospective observational cohort study	Patients with NSCLC undergoing radical lung cancer surgery	174	PLR; NLR	Preoperative complete blood count	High vs. low PLR (>148.6 vs. ≤148.6) and high vs. low NLR (>2.9 vs. ≤2.9)	Postoperative pulmonary complications, including pneumonia, bronchopleural fistula, respiratory failure, ARDS/ALI, atelectasis, prolonged air leak, and any pulmonary complication	Radical lung cancer surgery via thoracotomy (sublobectomy, lobectomy, bilobectomy, pneumonectomy, and sleeve lobectomy)	Age; sex; BMI; smoking history; ASA class; lung function; comorbidities; clinical symptoms; adjuvant therapy; resection type; tumor location; pathologic type; pathologic stage	Raw frequency data extractable; cut-off values available; AUC available for survival prediction
Miao et al. (10)	2024	China	Single-center retrospective cohort study	Adults undergoing elective lung resection surgery	386	SII; prealbumin	Preoperative	POP vs. no POP; continuous-variable logistic regression; combined SII-plus-prealbumin model	POP within 7 days after surgery	Segmentectomy or lobectomy via video-assisted thoracoscopic surgery	Age; sex; BMI; ASA class; smoking; drinking; hypertension; diabetes mellitus; coronary heart disease; COPD; perioperative clinical and laboratory variables	Adjusted ORs with 95% CIs; AUC and cut-off performance; continuous data extractable
Mizuno et al. (6)	2012	Japan	Retrospective cohort study	Patients with clinical idiopathic pulmonary fibrosis undergoing pulmonary resection for primary lung cancer	52	CRP	Preoperative	PAE vs. no PAE; multivariable logistic regression	Postoperative acute exacerbation of idiopathic pulmonary fibrosis after pulmonary resection	Segmentectomy, lobectomy, or bilobectomy via thoracotomy with thoracoscopic assistance	Preoperative CRP; pulmonary function; perioperative variables; fluid management variables	Adjusted OR with 95% CI available; raw event frequency extractable
Ruan et al. (11)	2025	China	Single-center retrospective cohort study	Patients with NSCLC undergoing lung resection	568 (252 after PSM analysis)	Neutrophil count; lymphocyte count; monocyte count; platelet count; NLR; PLR; LMR; SII	Within 3 days before surgery	POP vs. no POP after 1:3 propensity score matching; multivariable logistic regression; ROC analysis	POP and 1-month postoperative pulmonary imaging changes	Lung resection for NSCLC; VATS approach reported	Sex; age; BMI; smoking history; comorbidities; TNM stage; resection site; resection type; lymph node variables; operative variables; inflammatory biomarkers	Adjusted ORs with 95% CIs; AUC and cut-off performance; continuous data extractable
Shen et al. (12)	2017	China	Single-center retrospective cohort study	Patients with NSCLC undergoing lung cancer lobectomy	1058	NLR; WBC; A/G ratio; platelet; routine clinical variables	Within 3 days before surgery	POP vs. no POP; binary logistic regression for POP and ordinal logistic regression for POP severity	POP within 30 days after lobectomy; severity graded by the Clavien–Dindo classification	Lobectomy via VATS or open surgery	Age; sex; smoking history; ppoFEV1%; ppoDlco%; ASA class; CCI; BMI; stage; pathology; WBC; NLR; A/G ratio; platelet; surgical approach; blood loss; operative time	Adjusted ORs with 95% CIs; raw frequency data extractable

A/G, albumin/globulin ratio; ALI, acute lung injury; ARDS, acute respiratory distress syndrome; ASA, American Society of Anesthesiologists; AUC, area under the curve; BMI, body mass index; CCI, Charlson comorbidity index; CHD, coronary heart disease; CI, confidence interval; COPD, chronic obstructive pulmonary disease; CRP, C-reactive protein; CT, computed tomography; DLco, diffusing capacity of the lungs for carbon monoxide; ECMO, extracorporeal membrane oxygenation; FEV1, forced expiratory volume in 1 s; IPF, idiopathic pulmonary fibrosis; IQR, interquartile range; LMR, lymphocyte-to-monocyte ratio; NIV, non-invasive ventilation; NLR, neutrophil-to-lymphocyte ratio; NR, not reported; OLV, one-lung ventilation; OR, odds ratio; OT, open thoracotomy; PAE, postoperative acute exacerbation; PLR, platelet-to-lymphocyte ratio; POP, postoperative pneumonia; PPCs, postoperative pulmonary complications; ppoDlco%, postoperative predicted diffusion capacity for carbon monoxide of the lung; ppoFEV1%, postoperative predicted forced expiratory volume in 1 s; PSM, propensity score matching; ROC, receiver operating characteristic; SD, standard deviation; SII, systemic immune-inflammation index; VATS, video-assisted thoracoscopic surgery; WBC, white blood cell.

For Chang et al., adjusted PPC effect estimates were not directly available for the target inflammation-based biomarkers. For Lan et al., the reported cut-off values and AUCs were derived for survival prediction rather than PPC prediction. For Ruan et al., the study period was reported inconsistently in the abstract and Methods; this discrepancy is noted in the table. NR indicates not reported in the original article.

**Table 2:** Methodological quality of the included cohort studies assessed using the Newcastle–Ottawa scale (NOS).

Study	Year	Country	Type of Article	The Newcastle-Ottawa Scale (NOS)		
				Selection	Comparability	Outcome
Baar et al. (5)	2022	Germany	Retrospective multicenter cohort	* * * *	* *	* * *
Chang et al. (7)	2023	Taiwan	Retrospective cohort	* * * *	*	* * *
Jiang et al. (8)	2023	China	Retrospective cohort	* * * *	* *	* * *
Lan et al. (9)	2017	China	Prospective cohort	* * *	-	* * *
Miao et al. (10)	2024	China	Retrospective cohort	* * * *	* *	* * *
Mizuno et al. (6)	2012	Japan	Retrospective cohort	* * * *	*	* * *
Ruan et al. (11)	2025	China	Retrospective cohort	* * * *	* *	* * *
Shen et al. (12)	2017	China	Retrospective cohort	* * * *	* *	* * *

* = 1 point; maximum score=9 points.

NOS was applied using the cohort-study domains: Selection, Comparability, and Outcome.

"-” indicates that no star was awarded in that domain.

### Postoperative pulmonary complications (PPCs)

Eight studies were included in the systematic review (5–12). Because the included studies differed substantially in biomarker selection, effect metric, analytical scale, and pulmonary outcome definition, quantitative synthesis was considered secondary and exploratory. Several studies contributed more than one eligible estimate because they examined multiple biomarkers; in such instances, the estimates were considered separately for exploratory quantitative purposes, but interpretation remained anchored at the study level rather than the estimate level (7, 11).

An exploratory biomarker-specific pooled analysis was performed for preoperative neutrophil-to-lymphocyte ratio (NLR), including the univariable estimate reported by Chang et al. (7) and the multivariable-adjusted estimate reported by Shen et al. (12). The pooled association was not statistically significant under the random-effects model (OR 1.53, 95% CI 0.79–2.99; *P* = 0.21; Supplementary Figure S1), and heterogeneity was substantial (I^2^ = 96%). Although both studies showed effect estimates in the direction of increased postoperative pulmonary risk with higher NLR, the marked difference in effect size suggested limited comparability between the two studies (7, 12).

An exploratory pooled analysis including all extractable effect estimates across eligible biomarkers yielded an OR of 1.04 (95% CI 1.00–1.08; *P* = 0.07; Supplementary Figure S2), with substantial heterogeneity (I^2^ = 92%). Because this analysis combined different biomarkers, outcome subtypes, and, in some instances, more than one eligible estimate from the same source study, it was interpreted cautiously and was not regarded as a primary inferential analysis (5, 7–12).

To explore whether use of adjusted estimates reduced inconsistency, a sensitivity analysis restricted to adjusted effect estimates was performed. The pooled result remained non-significant (OR 1.01, 95% CI 0.97–1.04; *P* = 0.76; Supplementary Figure S3), and heterogeneity remained very high (I^2^ = 94%), suggesting that the observed inconsistency was not explained solely by adjustment status (5, 8, 10–12).

Because POP represented a major subtype within the PPC framework, an exploratory POP-only pooled analysis was also performed. When analyses were restricted to studies reporting POP, the pooled estimate was null (OR 1.00, 95% CI 0.97–1.03; *P* = 0.98; Supplementary Figure S4), with persistent substantial heterogeneity (I^2^ = 93%) (8, 10–12). Excluding the two adjusted estimates contributed by Ruan et al. (11), namely PLR and LMR, yielded a pooled OR of 1.26 (95% CI 0.80–1.98; *P* = 0.33; Supplementary Figure S5), but heterogeneity remained high (I^2^ = 93%) (8, 10, 12). Taken together, these findings suggest that the observed inconsistency was driven more by differences in biomarker handling and study design than by outcome definition alone.

A separate exploratory dichotomous meta-analysis was conducted using studies with extractable 2 × 2 data for cutoff-based biomarker categories, while avoiding double-counting of overlapping cohorts. In this analysis, higher preoperative biomarker levels were associated with increased PPC risk (OR 1.81, 95% CI 1.33–2.47; *P* = 0.0002; Supplementary Figure S6), with no heterogeneity detected (I^2^ = 0%) (5, 9). However, because this synthesis combined crude data across different biomarkers, it was interpreted as supportive rather than definitive evidence.

At the individual-study level, several biomarkers showed potentially adverse associations with postoperative pulmonary outcomes. In Baar et al. (5), elevated CRP (>3 mg/dL) was associated with increased PPC risk. In Jiang et al. (8) and Miao et al. (10), higher SII was associated with POP. In Shen et al. (12), higher NLR was independently associated with POP, whereas Chang et al. (7) showed a weaker, non-significant association for NLR. In Miao et al. (10), higher prealbumin appeared protective against POP. Ruan et al. (11) contributed two adjusted estimates, showing an inverse association for PLR and a stronger inverse association for LMR with POP; these directions differed from several of the other biomarker estimates and were therefore explored further in sensitivity analyses. Chang et al. (7) also reported additional estimates for PLR and albumin, but these findings were unstable or not sufficiently comparable for primary pooling. Mizuno et al. (6) was not included in the primary pooled analyses because it examined postoperative acute exacerbation of idiopathic pulmonary fibrosis rather than standard PPCs/POP in the general lung cancer surgery population, although it also suggested an adverse association between preoperative inflammation and postoperative pulmonary deterioration in a special high-risk subgroup.

At the individual-study level, CRP, SII, and NLR showed the most consistent adverse associations, whereas the findings for other biomarkers were more variable across studies.

## Discussion

In this systematic review, we found that the current literature linking preoperative inflammation-based biomarkers to postoperative pulmonary complications after lung cancer resection remains suggestive but not sufficiently standardized for a single pooled inference. Most included studies were retrospective, and substantial heterogeneity was present in biomarker selection, analytic scale, and pulmonary outcome definition. Under these conditions, the main value of the present review lies less in producing one summary effect size than in clarifying the structure of the available evidence. Across individual studies, CRP, SII, and NLR showed the most consistent adverse associations, whereas the findings for PLR, LMR, albumin, and prealbumin were more variable and appeared to be more context-dependent. The exploratory pooled analyses should therefore be read as supportive rather than definitive, with the most consistent message being that any clinically useful signal is likely to be biomarker-specific rather than uniform across all inflammation-based indices.

To interpret these findings appropriately, it is first necessary to consider why PPCs were used as the primary outcome framework. This choice was clinically appropriate. After lung resection, pulmonary morbidity is rarely limited to a single event; rather, it encompasses a spectrum of clinically relevant respiratory complications, including pneumonia, atelectasis, respiratory failure, and the need for postoperative ventilatory support. Contemporary perioperative literature has increasingly recognized PPCs as a meaningful composite framework because these events often share overlapping perioperative determinants, may coexist during the same postoperative course, and collectively influence recovery, length of stay, resource utilization, and early postoperative mortality (2, 15, 16). This broader framework was also justified by the structure of the available evidence in our review. Among the eight included studies, some investigators reported composite PPC endpoints, whereas others reported only specific pulmonary events, most commonly POP (5, 8, 10–12). Restricting the review to a single pulmonary event from the outset would therefore have excluded clinically informative cohorts and further reduced an already limited evidence base.

Within this framework, treating postoperative pneumonia as a major subtype was equally reasonable. POP is one of the most frequently reported and clinically consequential pulmonary complications after lung cancer surgery, and in practice it is often the most consistently defined single respiratory endpoint across observational thoracic cohorts (11, 17, 18). In our own synthesis, four studies contributed to the POP-only exploratory analysis (8, 10–12), underlining how often this endpoint was available even when broader PPC definitions varied. Recent external data further underscore its burden. In a 2025 retrospective cohort of patients undergoing lung surgery for cancer, POP occurred in 10.7% of cases, and bronchoscopy with bronchoalveolar lavage identified causative pathogens in 87.3% of POP cases (18). Other recent thoracic surgical work has likewise emphasized POP as a common pulmonary complication after lung resection and a practical target for perioperative risk stratification (17). Analyzing POP separately therefore did not conflict with the broader PPC framework; instead, it provided a more specific and comparable endpoint within a heterogeneous literature. In practical terms, this strategy allowed us to balance breadth and specificity: PPCs captured the overall burden of postoperative pulmonary morbidity, whereas POP provided the most recurrent and clinically interpretable subtype when direct cross-study comparison was possible.

Among the biomarkers evaluated in the present review, CRP, SII, and NLR appeared to show the most coherent signals of association with postoperative pulmonary morbidity. CRP was notable because it showed an adverse direction in both Baar et al. (5) and Mizuno et al. (6), despite the marked differences between those study populations. In the general lung cancer surgical cohort reported by Baar et al., preoperative CRP >3 mg/dL was associated with increased PPC risk, with an adjusted OR of 1.70 (95% CI 1.10–2.60). Although Mizuno et al. (6) studied a special high-risk subgroup with idiopathic pulmonary fibrosis rather than standard PPCs or POP, the direction of association was again unfavorable, suggesting that a preoperatively activated inflammatory state may be linked to greater vulnerability to postoperative pulmonary deterioration across different clinical settings.

SII also appeared relatively robust across the available evidence. In our review, Jiang et al. (8) and Miao et al. (10) both reported adverse associations between higher SII and POP. Miao et al. (10) observed an adjusted OR of 1.38 (95% CI 1.19–2.83), while Jiang et al., in a cohort of 1,486 patients, likewise identified preoperative SII as a predictor of postoperative pneumonia (8). This pattern is consistent with external thoracic surgical literature. In a 2022 study of 204 patients undergoing lung cancer resection, 40 patients developed 47 PPC events, and multivariable analysis identified SII together with FEV1 as the most important predictive factors (19). NLR likewise showed a meaningful signal, particularly in Shen et al. (12), where higher preoperative NLR was independently associated with POP with an adjusted OR of 2.171 (95% CI 1.721–2.737). However, the NLR signal was less stable across studies, because Chang et al. (7) showed only a weaker and non-significant association. By contrast, the evidence for PLR, LMR, albumin, and prealbumin remained more exploratory. For example, Ruan et al. (11) reported inverse adjusted associations for PLR and LMR, whereas Chang et al. (7) reported unstable estimates for PLR and albumin, and Miao et al. (10) suggested a protective association for prealbumin. Taken together, these data suggest that CRP, SII, and NLR are currently the most credible candidates for further validation, whereas the remaining biomarkers should be interpreted more cautiously and in a biomarker-specific rather than pooled manner.

The instability of the pooled results was not unexpected, because heterogeneity was present at multiple levels of the evidence base. First, the included studies did not evaluate a single inflammatory construct, but rather a series of partly overlapping biomarkers, including CRP (5, 6), SII (8, 10), NLR (7, 9, 12), PLR (7, 9, 11), LMR (11), albumin (7), and prealbumin (10). These markers do not capture exactly the same biological dimension and therefore should not be expected to behave as interchangeable predictors. Second, the studies also differed substantially in analytical scale and statistical handling. Some biomarkers were analyzed as continuous variables (7, 8, 10, 12), whereas others were dichotomized using study-specific cutoffs or modeled as high-vs.-low categories (5, 6, 9). Reported effect measures also varied, ranging from multivariable-adjusted odds ratios (5, 8, 10–12) to crude frequency data (5, 9) and predictive performance metrics such as AUC, sensitivity, and specificity (8, 10, 11). In prognosis research, such methodological diversity is a recognized source of between-study heterogeneity (20, 21). This is particularly relevant when continuous biomarkers are modeled differently, categorized using non-uniform thresholds, or reported as adjusted and unadjusted estimates that are not directly comparable (22, 23). In our review, this was evident numerically: the all-estimates synthesis remained highly heterogeneous (I^2^ = 92%), the adjusted-only synthesis remained similarly inconsistent (I^2^ = 94%), and even the biomarker-specific NLR synthesis, which included only two studies, showed I^2^ = 96% (7, 12). In addition, the Jiang et al. (8) estimate contributed substantial statistical weight in some exploratory models, likely because of the scale and modeling characteristics of the reported continuous SII effect estimate rather than because it was intrinsically more clinically informative than the other biomarkers. By contrast, the cutoff-based dichotomous synthesis based on extractable raw data from Baar et al. (5) and Lan et al. (9) yielded a materially different pooled estimate (OR 1.81, 95% CI 1.33–2.47; I^2^ = 0%), underscoring how strongly analytical structure influenced the pooled result.

Heterogeneity also arose from differences in outcome definition and patient population. Some studies reported composite PPC endpoints, whereas others focused specifically on POP, and one study evaluated postoperative acute exacerbation of idiopathic pulmonary fibrosis in a special high-risk subgroup rather than standard PPCs or POP in the general lung cancer surgery population (6). Even within the broader PPC framework, perioperative pulmonary outcomes are defined inconsistently across studies, and recent work on PPC prediction models has likewise identified heterogeneity in predicted outcomes and model structure as a major barrier to evidence synthesis and implementation (3, 15). The included cohorts also differed clinically with respect to surgical approach, extent of resection, baseline pulmonary risk, pathological characteristics, and study setting, ranging from a single prospective cohort (9) and several single-center retrospective cohorts (6–12) to a multicenter thoracic registry study (5). These differences were reflected in the POP-only synthesis, which remained null and highly heterogeneous (OR 1.00, 95% CI 0.97–1.03; I^2^ = 93%) (8, 10–12), and in the sensitivity analysis excluding the two estimates from Ruan et al. (11), where the pooled OR shifted to 1.26 (95% CI 0.80–1.98) but heterogeneity remained high (I^2^ = 93%) (8, 10, 12). Under these conditions, high I^2^ should not be viewed simply as a statistical nuisance. Rather, it reflects real conceptual and methodological inconsistency across the current literature.

Although the pooled estimates were unstable, this does not necessarily mean that the underlying clinical signal is absent. A plausible explanation is that elevated preoperative inflammation-based biomarkers may identify a host state that is already vulnerable before surgery. In lung cancer surgery, this vulnerability is unlikely to reflect a single process. Rather, it may include baseline systemic inflammatory activation, relative immune disequilibrium, increased susceptibility to perioperative infection, and reduced physiological or nutritional reserve. These pre-existing conditions can then be amplified by the perioperative insult itself. Lung resection is accompanied by surgical tissue injury, one-lung ventilation, alveolar collapse and re-expansion, and procedure-related pulmonary stress, all of which can intensify inflammatory signaling and impair postoperative pulmonary recovery (24, 25). Under these circumstances, a more activated inflammatory profile before surgery may plausibly predispose patients to pneumonia, atelectatic complications, respiratory failure, or delayed pulmonary recovery. This interpretation is also supported by the broader thoracic anesthesia and perioperative literature linking one-lung ventilation and perioperative inflammatory responses to postoperative pulmonary injury and PPC risk (24–26).

The biomarkers evaluated in the present review likely differ in what aspect of perioperative vulnerability they reflect. CRP may function as a relatively direct marker of baseline systemic inflammatory burden. NLR and SII may better capture the combined imbalance between innate inflammatory activation and relative suppression of adaptive immune competence, which may help explain why these indices showed more consistent adverse signals than some of the other markers (27, 28). SII may be particularly informative because it incorporates a platelet component in addition to neutrophils and lymphocytes, potentially reflecting a broader perioperative inflammatory phenotype. By contrast, albumin and prealbumin are less purely inflammatory and are more likely to reflect nutritional reserve, frailty, and tolerance to surgical stress. In lung cancer surgery, perioperative albumin decline has also been linked to PPC risk, and one retrospective cohort study reported that a postoperative albumin reduction cut-off of 14.97% identified patients at higher pulmonary risk after thoracoscopic anatomical lung cancer surgery (29). These markers should therefore not be viewed as interchangeable readouts of a single pathway, but rather as related yet distinct clinical signals within a broader perioperative risk profile.

Not all studies showed statistically significant associations, but this inconsistency should not be interpreted simply as evidence against biomarker relevance. A major explanation is methodological heterogeneity in how biomarkers were handled and analyzed. Some studies modeled biomarkers as continuous variables, whereas others used study-specific cutoffs or crude dichotomous comparisons. Such differences can materially affect both the magnitude and the apparent stability of effect estimates, particularly when continuous variables are categorized at arbitrary thresholds or entered into non-comparable regression frameworks (20, 22). This pattern was evident in the present dataset. For example, Shen et al. (12) reported an adjusted OR of 2.171 (95% CI 1.721–2.737) for NLR, whereas Chang et al. (7) reported only a weak and non-significant univariable association for NLR (OR 1.098, 95% CI 0.960–1.256), together with an unstable estimate for PLR (OR 1.078, 95% CI 0.272–5.841). This is unlikely to reflect a purely biological contradiction. More plausibly, it reflects differences in modeling strategy, case mix, and study purpose. Taken together, inconsistency across studies does not necessarily mean that preoperative inflammation-based biomarkers lack clinical value; rather, it suggests that their predictive contribution is likely to be context-dependent and easier to detect when biomarker handling and outcome definition are more consistent.

Clinical heterogeneity likely contributed as well. The included cohorts differed in surgical approach, extent of resection, background pulmonary reserve, smoking burden, and perioperative management, all of which may affect both baseline inflammatory status and the probability of postoperative pulmonary events. Recent work on PPC prediction models has likewise shown that heterogeneity in predicted outcomes, model structure, and target populations remains a major barrier to synthesis and implementation (3). This is directly relevant to the present review because some biomarkers may be more informative in settings with higher inflammatory or respiratory vulnerability than in lower-risk cohorts. The conflicting directions reported for some markers, particularly PLR and LMR in Ruan et al. (11), also illustrate how adjustment strategy and model specification can alter interpretation. More broadly, prognostic factor studies with limited event numbers, non-uniform modeling strategies, and variable design quality are known to yield unstable or non-replicable results (20, 23). Taken together, inconsistency across studies does not necessarily mean that preoperative inflammation-based biomarkers lack value. More likely, it indicates that their predictive contribution is context-dependent and becomes easier to detect when biomarker definition, outcome definition, case-mix, and adjustment strategy are more consistent.

Several included studies also reported predictive performance metrics such as AUC, cut-off values, sensitivity, and specificity, but these data were not sufficiently comparable for formal synthesis. The problem was not the absence of predictive signal, but the fact that these metrics were derived from different biomarkers, different thresholds, and different prediction frameworks. For example, Miao et al. (10) reported an AUC of 0.69 for SII alone and 0.60 for prealbumin alone, whereas the combined model reached an AUC of 0.79. By comparison, Ruan et al. (11) reported AUCs of 0.780 for PLR and 0.729 for LMR in relation to POP, with corresponding cutoffs of 140.315 and 4.875. Because these performance estimates were generated from different predictors and model structures, pooling them would create a misleading impression of uniformity. At present, they are better interpreted as descriptive evidence that some biomarkers may have discriminative potential rather than as directly combinable measures of one standardized clinical tool.

More broadly, these findings suggest that this field remains at an early stage of predictive exploration rather than at the stage of a standardized and directly generalizable clinical tool. The same applies to severity- or recovery-related outcomes. In the current evidence base, these outcomes were too sparse and too inconsistently defined to support formal synthesis. For example, Ruan et al. (11) extended the analysis beyond POP occurrence to one-month postoperative CT recovery, classifying patients as worse, unchanged, or improved, but no significant differences were observed across biomarker-defined risk groups (*P* = 0.976). This illustrates the broader problem: severity and recovery outcomes were not reported using a common framework and did not represent the same construct across studies. Consistent with our prespecified strategy, these data were therefore summarized descriptively. At present, the available AUC, cut-off, and recovery data are useful for hypothesis generation and local model development, but they do not yet support a standardized, directly implementable clinical prediction tool.

Despite these limitations, the present review has several strengths. First, it addressed a focused and clinically relevant question in a setting where preoperative pulmonary risk assessment remains difficult despite advances in perioperative care. By restricting the population to patients undergoing lung resection for lung cancer and prespecifying PPCs as the primary outcome framework, we reduced the heterogeneity that would have arisen from mixing other thoracic procedures or non-pulmonary postoperative endpoints. Second, the review was prospectively registered and conducted in accordance with PRISMA 2020 and Cochrane methodological guidance (13, 14), with predefined eligibility criteria, duplicate study selection and data extraction, and structured quality assessment. Third, the synthesis strategy was matched to the structure of the available evidence. Rather than forcing a single pooled estimate across clearly dissimilar biomarkers and data types, we separated effect-estimate-based analyses from crude dichotomous analyses, prioritized adjusted estimates where available, treated POP as a major PPC subtype, and interpreted non-independent estimates cautiously. Finally, we preserved clinical meaning at the study level by retaining methodologically distinct but clinically informative evidence, including the special high-risk IPF subgroup, within the qualitative synthesis (6).

Several limitations should also be acknowledged. First, the evidence base was small and consisted predominantly of retrospective cohort studies, especially single-center retrospective cohorts (5–8, 10–12), which increases the risk of selection bias, residual confounding, and instability of effect estimates. Second, there was substantial heterogeneity across studies in biomarker choice, cut-off definition, analytical scale, and pulmonary endpoint reporting (5–12). Third, although adjusted estimates were prioritized when available, multivariable estimates were not uniformly available for all target biomarkers, and some exploratory syntheses necessarily included a mixture of adjusted, univariable, and crude data structures (5, 7–12). Fourth, several studies contributed more than one eligible estimate, and although these were interpreted cautiously at the study level, complete statistical independence could not always be maintained in exploratory syntheses (7, 11). Fifth, one included study evaluated postoperative acute exacerbation of idiopathic pulmonary fibrosis rather than standard PPCs or POP in the general lung cancer surgery population and therefore contributed only to qualitative synthesis (6). Finally, because fewer than 10 studies were available for any pooled analysis, formal assessment of publication bias was not feasible. These limitations mean that the present findings should be interpreted as a structured synthesis of an emerging literature rather than as definitive pooled evidence for immediate clinical adoption.

In summary, the current evidence suggests that selected preoperative inflammation-based biomarkers, particularly CRP, SII, and NLR, may be associated with postoperative pulmonary morbidity after lung resection for lung cancer. However, the available literature remains biomarker-specific, dominated by retrospective cohort studies, and marked by substantial methodological heterogeneity. At present, these markers are better regarded as candidates for further validation than as ready-to-use clinical tools. Future prospective multicenter studies with standardized biomarker definitions, more consistent pulmonary outcome reporting, and biomarker-specific analytical strategies will be needed to determine how these markers should be incorporated into perioperative risk stratification.

## Data Availability

The data analyzed in this study is subject to the following licenses/restrictions: all data generated or analyzed during this study are included in this published article and its Supplementary Material files. Additional details are available from the corresponding author upon reasonable request. Requests to access these datasets should be directed to vicky19801979@sina.com.

## Supplementary material

Supplementary Figure S1 Exploratory biomarker-specific pooled analysis for neutrophil-to-lymphocyte ratio (NLR), presented as a random-effects forest plot. Two eligible NLR estimates were included, one from Chang 2023 and one from Shen 2017. The pooled association was not statistically significant, and heterogeneity was substantial. NLR, neutrophil-to-lymphocyte ratio; PPCs, postoperative pulmonary complications; POP, postoperative pneumonia; OR, odds ratio; CI, confidence interval. Footnote: Chang 2023 = NLR; Shen 2017 = NLR.

Supplementary Figure S2 Exploratory pooled analysis including all eligible extractable effect estimates, presented as a random-effects forest plot. This exploratory analysis included all extractable eligible estimates across different preoperative inflammation-based biomarkers and pulmonary outcome subtypes. It should be interpreted cautiously because different biomarkers, analytical scales, and more than one estimate from the same study were combined. PPCs, postoperative pulmonary complications; POP, postoperative pneumonia; OR, odds ratio; CI, confidence interval; CRP, C-reactive protein; PLR, platelet-to-lymphocyte ratio; SII, systemic immune-inflammation index; LMR, lymphocyte-to-monocyte ratio. Footnote: Baar 2022 = CRP >3 mg/dL; Chang 2023 = NLR; Chang 2023* = PLR; Chang 2023** = albumin ≤3.5 g/L; Jiang 2023 = SII; Lan 2017 = NLR >2.9; Miao 2024 = SII; Miao 2024* = prealbumin; Ruan 2025 = PLR; Ruan 2025* = LMR; Shen 2017 = NLR.

Supplementary Figure S3 Sensitivity analysis restricted to adjusted effect estimates, presented as a random-effects forest plot. Only studies contributing adjusted effect estimates were retained in this analysis. The pooled result remained non-significant, and heterogeneity remained substantial. PPCs, postoperative pulmonary complications; OR, odds ratio; CI, confidence interval; CRP, C-reactive protein; SII, systemic immune-inflammation index; PLR, platelet-to-lymphocyte ratio; LMR, lymphocyte-to-monocyte ratio; NLR, neutrophil-to-lymphocyte ratio. Footnote: Baar 2022 = CRP >3 mg/dL; Jiang 2023 = SII; Miao 2024 = SII; Ruan 2025 = PLR; Ruan 2025* = LMR; Shen 2017 = NLR.

Supplementary Figure S4 Exploratory pooled analysis restricted to postoperative pneumonia (POP), presented as a random-effects forest plot. This analysis was limited to studies reporting POP, which was treated as a major subtype within the postoperative pulmonary complications framework. The pooled estimate was null, and heterogeneity remained substantial. POP, postoperative pneumonia; PPCs, postoperative pulmonary complications; OR, odds ratio; CI, confidence interval; SII, systemic immune-inflammation index; PLR, platelet-to-lymphocyte ratio; LMR, lymphocyte-to-monocyte ratio; NLR, neutrophil-to-lymphocyte ratio. Footnote: Jiang 2023 = SII; Miao 2024 = SII; Miao 2024* = prealbumin; Ruan 2025 = PLR; Ruan 2025* = LMR; Shen 2017 = NLR.

Supplementary Figure S5 Sensitivity analysis of the POP-only pooled analysis after exclusion of the two estimates from Ruan 2025, presented as a random-effects forest plot. This sensitivity analysis excluded the two adjusted estimates contributed by Ruan 2025 to examine their influence on the POP-only synthesis. The pooled estimate remained non-significant, and heterogeneity persisted. POP, postoperative pneumonia; OR, odds ratio; CI, confidence interval; SII, systemic immune-inflammation index; NLR, neutrophil-to-lymphocyte ratio; PLR, platelet-to-lymphocyte ratio; LMR, lymphocyte-to-monocyte ratio. Footnote: Jiang 2023 = SII; Miao 2024 = SII; Miao 2024* = prealbumin; Shen 2017 = NLR. Deleted estimates: Ruan 2025 = PLR; Ruan 2025* = LMR.

Supplementary Figure S6 Exploratory dichotomous meta-analysis of cutoff-based biomarker categories after avoidance of double-counting of the Lan cohort, presented as a fixed-effect forest plot. This exploratory dichotomous analysis was restricted to studies with extractable 2 × 2 data for cutoff-based biomarker categories. To avoid double-counting, only one eligible comparison from Lan 2017 was retained. Higher preoperative biomarker levels were associated with increased risk of postoperative pulmonary complications. PPCs, postoperative pulmonary complications; OR, odds ratio; CI, confidence interval; CRP, C-reactive protein; PLR, platelet-to-lymphocyte ratio. Footnote: Baar 2022 = CRP >3 mg/dL; Lan 2017 = PLR >148.6 vs. ≤148.6.

## References

[1] Bray F Laversanne M Sung H Ferlay J Siegel RL Soerjomataram I. Global cancer statistics 2022: GLOBOCAN estimates of incidence and mortality worldwide for 36 cancers in 185 countries. CA Cancer J Clin. (2024) 74(3):229–63. 10.3322/caac.21834 38572751

[2] Batchelor TJP Rasburn NJ Abdelnour-Berchtold E Brunelli A Cerfolio RJ Gonzalez M. Guidelines for enhanced recovery after lung surgery: recommendations of the enhanced recovery after surgery (ERAS®) society and the European society of thoracic surgeons (ESTS). Eur J Cardiothorac Surg. (2019) 55(1):91–115. 10.1093/ejcts/ezy301 30304509

[3] Huang Z Han Y Zhuang H Jiang J Zhou C Yu H. Prediction models for postoperative pulmonary complications: a systematic review and meta-analysis. Br J Anaesth. (2025) 135(5):1415–27. 10.1016/j.bja.2025.04.025 40473567 PMC12597335

[4] Dyas AR Stuart CM Bronsert MR Kelleher AD Bata KE Cumbler EU. Anatomic lung resection outcomes after implementation of a universal thoracic ERAS protocol across a diverse health care system. Ann Surg. (2024) 279(6):1062–9. 10.1097/SLA.0000000000006243 38385282 PMC11087203

[5] Baar W Semmelmann A Knoerlein J Weber F Heinrich S Loop T. Loop T; working group of the German thorax registry. Risk factors for postoperative pulmonary complications leading to increased in-hospital mortality in patients undergoing thoracotomy for primary lung cancer resection: a multicentre retrospective cohort study of the German thorax registry. J Clin Med. (2022) 11(19):5774. 10.3390/jcm11195774 36233649 PMC9572507

[6] Mizuno Y Iwata H Shirahashi K Takamochi K Oh S Suzuki K. The importance of intraoperative fluid balance for the prevention of postoperative acute exacerbation of idiopathic pulmonary fibrosis after pulmonary resection for primary lung cancer. Eur J Cardiothorac Surg. (2012) 41(6):e161–5. 10.1093/ejcts/ezs147 22504895

[7] Chang BS Peng TC Lue KH Wu YF Huang CH. Comprehensive assessment of the clinical risk factors of postoperative adverse events and survival in patients with non-small-cell lung cancer. In Vivo. (2023) 37(3):1358–64. 10.21873/invivo.13217 37103097 PMC10188011

[8] Jiang R Li P Shen W Deng H Qin C Qiu X. The predictive value of the preoperative systemic immune-inflammation index in the occurrence of postoperative pneumonia in non-small cell lung cancer: a retrospective study based on 1486 cases. Thorac Cancer. (2023) 14(1):30–5. 10.1111/1759-7714.14691 36495040 PMC9807440

[9] Lan H Zhou L Chi D Zhou Q Tang X Zhu D. Preoperative platelet to lymphocyte and neutrophil to lymphocyte ratios are independent prognostic factors for patients undergoing lung cancer radical surgery: a single institutional cohort study. Oncotarget. (2017) 8(21):35301–10. 10.18632/oncotarget.13312 27845912 PMC5471056

[10] Miao H Ge D Wang Q Zhou L Chen H Qin Y. Predictive significance of systemic immune-inflammation index combined with prealbumin for postoperative pneumonia following lung resection surgery. BMC Pulm Med. (2024) 24(1):277. 10.1186/s12890-024-03086-7 38862955 PMC11167804

[11] Ruan Y Cao W Han J Yang A Xu J Zhang T. Impact of preoperative inflammatory biomarkers on postoperative pneumonia and one-month pulmonary imaging changes after surgery for non-small cell lung cancer. Front Oncol. (2025) 15:1489068. 10.3389/fonc.2025.1489068 40171252 PMC11958979

[12] Shen Q Lai Y Li X Ji Y Zhou K. The role of an array of routine clinical variables to the occurrence and severity of postoperative pneumonia in non–small cell lung cancer patients. Int Surg. (2017) 102:396–403. 10.9738/INTSURG-D-16-00193.1

[13] Page MJ McKenzie JE Bossuyt PM Boutron I Hofmann TC Mulrow CD. The PRISMA 2020 statement: an updated guideline for reporting systematic reviews. Br Med J. (2021) 372:n71. 10.1136/bmj.n71 33782057 PMC8005924

[14] Higgins JPT Thomas J Chandler J Cumpston M Li T Page MJ. Cochrane Handbook for Systematic Reviews of Interventions Version 6.3 (updated February 2022). Chichester: Cochrane (2022). Available online at: https://training.cochrane.org/handbook/current

[15] Abbott TEF Fowler AJ Pelosi P Gama de Abreu M Møller AM Canet J. Pearse RM; StEP-COMPAC group. A systematic review and consensus definitions for standardised end-points in perioperative medicine: pulmonary complications. Br J Anaesth. (2018) 120(5):1066–79. 10.1016/j.bja.2018.02.007 29661384

[16] Semmelmann A Baar W Fellmann N Moneke I Loop T, Working Group of the German Thorax Registry. The impact of postoperative pulmonary complications on perioperative outcomes in patients undergoing pneumonectomy: a multicenter retrospective cohort study of the German thorax registry. J Clin Med. (2023) 13(1):35. 10.3390/jcm13010035 38202042 PMC10779566

[17] Verkoulen KCHA Laven IEWG Daemen JHT Degens JHRJ Hendriks LEL Hulsewé KWE. The (un)lucky seven-how can we mitigate risk factors for postoperative pneumonia after lung resections? Transl Lung Cancer Res. (2024) 13(8):1763–7. 10.21037/tlcr-24-428 39263029 PMC11384492

[18] Giudici VM Brascia D Mangiameli G Voulaz E Bottoni E Crepaldi A. Identifying risk factors and pathogens in postoperative pneumonia after lung surgery for cancer: a retrospective cohort study. J Thorac Dis. (2025) 17(9):6530–41. 10.21037/jtd-2025-169 41158357 PMC12557710

[19] Xiaowei M Wei Z Qiang W Yiqian N Yanjie N Liyan J. Assessment of systemic immune-inflammation index in predicting postoperative pulmonary complications in patients undergoing lung cancer resection. Surgery. (2022) 172(1):365–70. 10.1016/j.surg.2021.12.023 35101329

[20] Riley RD Moons KGM Snell KIE Ensor J Hooft L Altman DG. A guide to systematic review and meta-analysis of prognostic factor studies. Br Med J. (2019) 364:k4597. 10.1136/bmj.k4597 30700442

[21] Damen JAA Moons KGM Van Smeden M Hooft L. How to conduct a systematic review and meta-analysis of prognostic model studies. Clin Microbiol Infect. (2023) 29(4):434–40. 10.1016/j.cmi.2022.07.019 35934199 PMC9351211

[22] Polley MC Dignam JJ. Statistical considerations in the evaluation of continuous biomarkers. J Nucl Med. (2021) 62(5):605–11. 10.2967/jnumed.120.251520 33579807 PMC8844257

[23] Sekula P Steinbrenner I Schultheiss UT Valveny N Rebora P Halabi S. Gail MH; for topic group 5 of the STRATOS initiative. Design aspects for prognostic factor studies. BMJ Open. (2025) 15(8):e095065. 10.1136/bmjopen-2024-095065 40887130 PMC12406931

[24] Furák J Németh T Lantos J Fabó C Géczi T Zombori-Tóth N. Perioperative systemic inflammation in lung cancer surgery. Front Surg. (2022) 9:883322. 10.3389/fsurg.2022.883322 35669251 PMC9163434

[25] De La Gala F Piñeiro P Reyes A Vara E Olmedilla L Cruz P. Postoperative pulmonary complications, pulmonary and systemic inflammatory responses after lung resection surgery with prolonged one-lung ventilation. Randomized controlled trial comparing intravenous and inhalational anaesthesia. Br J Anaesth. (2017) 119(4):655–63. 10.1093/bja/aex230 29121283

[26] Piccioni F Langiano N Bignami E Guarnieri M Proto P D'Andrea R. One-Lung ventilation and postoperative pulmonary complications after Major lung resection surgery. A multicenter randomized controlled trial. J Cardiothorac Vasc Anesth. (2023) 37(12):2561–71. 10.1053/j.jvca.2023.04.029 37730455 PMC10133024

[27] Jaillon S Ponzetta A Di Mitri D Santoni A Bonecchi R Mantovani A. Neutrophil diversity and plasticity in tumour progression and therapy. Nat Rev Cancer. (2020) 20(9):485–503. 10.1038/s41568-020-0281-y 32694624

[28] Cupp MA Cariolou M Tzoulaki I Aune D Evangelou E Berlanga-Taylor AJ. Neutrophil to lymphocyte ratio and cancer prognosis: an umbrella review of systematic reviews and meta-analyses of observational studies. BMC Med. (2020) 18(1):360. 10.1186/s12916-020-01817-1 33213430 PMC7678319

[29] Li P Li J Lai Y Wang Y Wang X Su J. Perioperative changes of serum albumin are a predictor of postoperative pulmonary complications in lung cancer patients: a retrospective cohort study. J Thorac Dis. (2018) 10(10):5755–63. 10.21037/jtd.2018.09.113 30505483 PMC6236178

