# Supplementary material for: Preoperative inflammation-based biomarkers and postoperative pulmonary complications after lung resection for lung cancer: a systematic review with selective exploratory quantitative synthesis
Source: Front Surg. 2026 Jul 30;13:1853516. doi: 10.3389/fsurg.2026.1853516 (PMC13469261; doi:10.3389/fsurg.2026.1853516)
Supplement: Supplementary file 1 [file Table1.docx]

**Supplementary Methods: Literature search strategy**

We systematically searched PubMed, Embase, Web of Science Core Collection, Scopus, and the Cochrane Library to identify studies evaluating the association of preoperative inflammation-based biomarkers with postoperative pulmonary complications after lung resection for lung cancer. The searches were performed from database inception to 20 March 2026. In addition, the reference lists of all included studies and relevant reviews were manually screened to identify any further eligible studies. The detailed search strategies for each database are provided below.

**1. PubMed**

**Database:** PubMed (MEDLINE)

**Date range:** from database inception to 20 March 2026

**Search field:** Title/Abstract and related indexing terms

Search strategy:

((("lung cancer" OR "lung neoplasm*" OR "lung carcinoma*" OR "lung tumor*" OR "pulmonary neoplasm*" OR "pulmonary carcinoma*" OR "non-small cell lung cancer" OR "non-small-cell lung cancer" OR NSCLC OR "lung adenocarcinoma" OR "lung squamous cell carcinoma"))

AND

("lung resection" OR "pulmonary resection" OR "lung surgery" OR "thoracic surgery" OR lobectom* OR segmentectom* OR "wedge resection" OR pneumonectom* OR bilobectom* OR "lung cancer surgery")

AND

(preoperativ* OR presurg* OR "before surgery" OR "before operation")

AND

("inflammation-based biomarker*" OR "inflammatory biomarker*" OR "inflammatory marker*" OR "inflammation-based index" OR "systemic inflammatory index" OR "systemic immune-inflammation index" OR SII OR NLR OR "neutrophil-to-lymphocyte ratio" OR PLR OR "platelet-to-lymphocyte ratio" OR LMR OR "lymphocyte-to-monocyte ratio" OR SIRI OR "systemic inflammation response index" OR PIV OR "pan-immune inflammation value" OR PNI OR "prognostic nutritional index" OR AISI OR "aggregate index of systemic inflammation" OR WBC OR "white blood cell*" OR neutrophil* OR lymphocyte* OR monocyte* OR RDW OR "red cell distribution width" OR CRP OR "C-reactive protein" OR albumin OR prealbumin OR "C-reactive protein-to-albumin ratio" OR CAR)

AND

("postoperative pulmonary complication*" OR PPC OR PPCs OR "postoperative pulmonary morbidity" OR "pulmonary complication*" OR "postoperative pneumonia" OR "postoperative pulmonary infection" OR "post-surgical pneumonia" OR "postoperative lung infection" OR atelectasis OR "prolonged air leak" OR "respiratory failure" OR empyema OR "pleural effusion"))

NOT

(review OR "systematic review" OR meta-analysis OR metaanalysis OR editorial OR letter OR "case report" OR "case reports" OR "conference abstract" OR "meeting abstract" OR "oral presentation" OR poster)

**2. Embase**

**Database:** Embase

**Date range:** from database inception to 20 March 2026

**Search strategy:**

('lung cancer' OR 'lung neoplasm*' OR 'lung carcinoma*' OR 'lung tumor*' OR 'pulmonary neoplasm*' OR 'pulmonary carcinoma*' OR 'non-small cell lung cancer' OR 'non-small-cell lung cancer' OR NSCLC OR 'lung adenocarcinoma' OR 'lung squamous cell carcinoma')

AND

('lung resection' OR 'pulmonary resection' OR 'lung surgery' OR 'thoracic surgery' OR lobectom* OR segmentectom* OR 'wedge resection' OR pneumonectom* OR bilobectom* OR 'lung cancer surgery')

AND

(preoperativ* OR presurg* OR 'before surgery' OR 'before operation')

AND

('inflammation-based biomarker*' OR 'inflammatory biomarker*' OR 'inflammatory marker*' OR 'inflammation-based index' OR 'systemic inflammatory index' OR 'systemic immune-inflammation index' OR SII OR NLR OR 'neutrophil-to-lymphocyte ratio' OR PLR OR 'platelet-to-lymphocyte ratio' OR LMR OR 'lymphocyte-to-monocyte ratio' OR SIRI OR 'systemic inflammation response index' OR PIV OR 'pan-immune inflammation value' OR PNI OR 'prognostic nutritional index' OR AISI OR 'aggregate index of systemic inflammation' OR WBC OR 'white blood cell*' OR neutrophil* OR lymphocyte* OR monocyte* OR RDW OR 'red cell distribution width' OR CRP OR 'C-reactive protein' OR albumin OR prealbumin OR 'C-reactive protein-to-albumin ratio' OR CAR)

AND

('postoperative pulmonary complication*' OR PPC OR PPCs OR 'postoperative pulmonary morbidity' OR 'pulmonary complication*' OR 'postoperative pneumonia' OR 'postoperative pulmonary infection' OR 'post-surgical pneumonia' OR 'postoperative lung infection' OR atelectasis OR 'prolonged air leak' OR 'respiratory failure' OR empyema OR 'pleural effusion')

NOT

('review' OR 'systematic review' OR 'meta analysis' OR 'editorial' OR 'letter' OR 'case report' OR 'conference abstract' OR 'meeting abstract' OR 'oral presentation' OR poster)

**3. Web of Science Core Collection**

**Database:** Web of Science Core Collection

**Date range:** from database inception to 20 March 2026

**Search field:** Topic (TS)

**Search strategy:**

TS=('lung cancer' OR 'lung neoplasm*' OR 'lung carcinoma*' OR 'lung tumor*' OR 'pulmonary neoplasm*' OR 'pulmonary carcinoma*' OR 'non-small cell lung cancer' OR 'non-small-cell lung cancer' OR NSCLC OR 'lung adenocarcinoma' OR 'lung squamous cell carcinoma')

AND

('lung resection' OR 'pulmonary resection' OR 'lung surgery' OR 'thoracic surgery' OR lobectom* OR segmentectom* OR 'wedge resection' OR pneumonectom* OR bilobectom* OR 'lung cancer surgery')

AND

(preoperativ* OR presurg* OR 'before surgery' OR 'before operation')

AND

('inflammation-based biomarker*' OR 'inflammatory biomarker*' OR 'inflammatory marker*' OR 'inflammation-based index' OR 'systemic inflammatory index' OR 'systemic immune-inflammation index' OR SII OR NLR OR 'neutrophil-to-lymphocyte ratio' OR PLR OR 'platelet-to-lymphocyte ratio' OR LMR OR 'lymphocyte-to-monocyte ratio' OR SIRI OR 'systemic inflammation response index' OR PIV OR 'pan-immune inflammation value' OR PNI OR 'prognostic nutritional index' OR AISI OR 'aggregate index of systemic inflammation' OR WBC OR 'white blood cell*' OR neutrophil* OR lymphocyte* OR monocyte* OR RDW OR 'red cell distribution width' OR CRP OR 'C-reactive protein' OR albumin OR prealbumin OR 'C-reactive protein-to-albumin ratio' OR CAR)

AND

('postoperative pulmonary complication*' OR PPC OR PPCs OR 'postoperative pulmonary morbidity' OR 'pulmonary complication*' OR 'postoperative pneumonia' OR 'postoperative pulmonary infection' OR 'post-surgical pneumonia' OR 'postoperative lung infection' OR atelectasis OR 'prolonged air leak' OR 'respiratory failure' OR empyema OR 'pleural effusion')

NOT

('review' OR 'systematic review' OR 'meta analysis' OR 'editorial' OR 'letter' OR 'case report' OR 'conference abstract' OR 'meeting abstract' OR 'oral presentation' OR poster)

**4. Scopus**

**Database:** Scopus

**Date range:** from database inception to 20 March 2026

**Search field:** TITLE-ABS-KEY

**Search strategy:**

TITLE-ABS-KEY( ( "lung cancer" OR "lung neoplasm*" OR "lung carcinoma*" OR "lung tumor*" OR "non-small cell lung cancer" OR "non-small-cell lung cancer" OR NSCLC OR "lung adenocarcinoma" OR "lung squamous cell carcinoma")

AND

( "lung resection" OR "pulmonary resection" OR "lung surgery" OR "thoracic surgery"

OR lobectom* OR segmentectom* OR "wedge resection" OR pneumonectom* OR bilobectom* OR "lung cancer surgery" )

AND

(preoperativ* OR presurg* OR "before surgery" OR "before operation")

AND

( SII OR NLR OR PLR OR LMR OR SIRI OR PIV OR PNI OR AISI OR WBC OR RDW OR CRP OR albumin OR prealbumin OR CAR OR "systemic immune-inflammation index" OR "neutrophil-to-lymphocyte ratio" OR "platelet-to-lymphocyte ratio" OR "lymphocyte-to-monocyte ratio" OR "systemic inflammation response index" OR "pan-immune inflammation value" OR "prognostic nutritional index" OR "red cell distribution width" OR "C-reactive protein" OR neutrophil* OR lymphocyte* OR monocyte*)

AND

("postoperative pulmonary complication*" OR PPC OR PPCs OR "pulmonary complication*" OR "postoperative pneumonia" OR "postoperative pulmonary infection"

OR atelectasis OR "prolonged air leak" OR "respiratory failure" OR empyema OR "pleural effusion"))

AND NOT TITLE-ABS-KEY(review OR "systematic review" OR "meta analysis" OR editorial OR letter OR "case report" OR "conference abstract" OR "meeting abstract"

OR "oral presentation" OR poster)

**5. Cochrane Library**

**Database:** Cochrane Library

**Date range:** from database inception to 20 March 2026

**Search strategy:**

(('lung cancer' OR 'lung neoplasm*' OR 'lung carcinoma*' OR 'lung tumor*' OR 'pulmonary neoplasm*' OR 'pulmonary carcinoma*' OR 'non-small cell lung cancer' OR 'non-small-cell lung cancer' OR NSCLC OR 'lung adenocarcinoma' OR 'lung squamous cell carcinoma')

AND

('lung resection' OR 'pulmonary resection' OR 'lung surgery' OR 'thoracic surgery' OR lobectom* OR segmentectom* OR 'wedge resection' OR pneumonectom* OR bilobectom* OR 'lung cancer surgery')

AND

(preoperativ* OR presurg* OR 'before surgery' OR 'before operation')

AND

('inflammation-based biomarker*' OR 'inflammatory biomarker*' OR 'inflammatory marker*' OR 'inflammation-based index' OR 'systemic inflammatory index' OR 'systemic immune-inflammation index' OR SII OR NLR OR 'neutrophil-to-lymphocyte ratio' OR PLR OR 'platelet-to-lymphocyte ratio' OR LMR OR 'lymphocyte-to-monocyte ratio' OR SIRI OR 'systemic inflammation response index' OR PIV OR 'pan-immune inflammation value' OR PNI OR 'prognostic nutritional index' OR AISI OR 'aggregate index of systemic inflammation' OR WBC OR 'white blood cell*' OR neutrophil* OR lymphocyte* OR monocyte* OR RDW OR 'red cell distribution width' OR CRP OR 'C-reactive protein' OR albumin OR prealbumin OR 'C-reactive protein-to-albumin ratio' OR CAR)

AND

('postoperative pulmonary complication*' OR PPC OR PPCs OR 'postoperative pulmonary morbidity' OR 'pulmonary complication*' OR 'postoperative pneumonia' OR 'postoperative pulmonary infection' OR 'post-surgical pneumonia' OR 'postoperative lung infection' OR atelectasis OR 'prolonged air leak' OR 'respiratory failure' OR empyema OR 'pleural effusion'))
