# Supplementary material for: Preoperative inflammation-based biomarkers and postoperative pulmonary complications after lung resection for lung cancer: a systematic review with selective exploratory quantitative synthesis
Source: Front Surg. 2026 Jul 30;13:1853516. doi: 10.3389/fsurg.2026.1853516 (PMC13469261; doi:10.3389/fsurg.2026.1853516)
Supplement: Supplementary file 2 [file Table2.docx]

| Supplementary Table S1. Detailed data extraction and study characteristics of the included studies | | | | | | | | | | | | | | | | | | | | | | | | | | | | | | | | | | | | | |  |
| --- | --- | --- | --- | --- | --- | --- | --- | --- | --- | --- | --- | --- | --- | --- | --- | --- | --- | --- | --- | --- | --- | --- | --- | --- | --- | --- | --- | --- | --- | --- | --- | --- | --- | --- | --- | --- | --- | --- |
| Authors | Year | Country | Study design | Study period | Setting / center | Population / disease entity | Eligibility / key exclusions | Total (n) | Male (n) | Female (n) | Age overall | Smoking history | Histology overall | Stage range / pathological stage | Surgical procedure | Surgical approach | Biomarker(s) assessed | Biomarker definition / formula | Specimen type | Timing of biomarker measurement | Biomarker cut-off(s) | Comparator / exposure model | PPC outcome framework | Exact pulmonary outcomes reported | Outcome ascertainment window | No. with PPC / POP / outcome event | Main clinicopathological variables reported | Univariable data available | Multivariable analysis performed | Adjusted variables | Effect estimate type | OR / RR / HR | 95% CI | AUC | Sensitivity / specificity / cut-off performance | Continuous data extractable | Raw frequency data extractable | |
| Baar et al. [5] | 2022 | Germany | Multicenter retrospective cohort study | 2016 to 2020 | German Thorax Registry; four participating centers | Patients undergoing open thoracotomy lung resection for primary lung cancer | Screened population: patients undergoing thoracotomy for non-cardiac surgery; analytic cohort: patients undergoing open thoracotomy lung resection for primary lung cancer; excluded indications included metastatic tumors, benign tumors, and mediastinal tumors | 1426 | NR | NR | NR | Never/current/former (cessation ≥3 months) reported | Primary lung cancer histology not separately reported | Primary lung cancer; detailed stage distribution not reported | Pneumonectomy, lobectomy, bilobectomy, segmentectomy, wedge resection, and bronchoplasty | Open thoracotomy | CRP; FEV1 | CRP >3 mg/dL; FEV1 <60% | Peripheral blood / routine preoperative assessment | Preoperative | CRP >3 mg/dl; FEV1 <60% | PPC versus no PPC; multivariable stepwise logistic regression | Composite PPC outcome | PPC components: prolonged air leak ≥7 days, postoperative pneumonia, NIV due to respiratory failure, new chest drain after surgery, re-intubation/continued ventilation after surgery, pleural empyema, ECMO | In-hospital period / index hospitalization | 472 patients with ≥1 PPC; component counts: prolonged air leak 235, postoperative pneumonia 227, NIV 84, new chest drain 62, re-intubation/continued ventilation 36, pleural empyema 14, ECMO 2 | Sex; age; BMI; smoking history; ASA class; neoadjuvant therapy; pulmonary infection within 4 weeks before surgery; CRP; hemoglobin; pH; paO2; paCO2; FEV1; operative variables; anesthetic variables | Yes | Yes | Sex; age ≥60 years; current or former smoking; CRP >3 mg/dL; FEV1 <60%; duration of surgery ≥195 min; intraoperative blood loss; partial ligation of the pulmonary artery; PRBC transfusion; OLV PEEP ≤5 cmH2O; OLV duration ≥175 min; epidural/paravertebral analgesia; crystalloid infusion ≥6 mL/kg/h; postoperative mechanical ventilation | OR | CRP >3 mg/dl: 1.7; FEV1 <60%: 1.4 | CRP >3 mg/dl: 1.1–2.6; FEV1 <60%: 1.0–1.9 | NR | NR | No | Yes | |
| Chang et al. [7] | 2023 | Taiwan | Single-center retrospective cohort study | December 2008 to December 2019 | Hualien Tzu Chi General Hospital / Hualien Tzu Chi Hospital; single academic medical center | Patients with resectable stage I-IIIA NSCLC undergoing curative-intent surgery | Included: age ≥18 years, VATS or open thoracotomy with curative intent, no active infection or infection resolved before therapy; excluded: previous cancer, previous lung surgery, uncontrolled concurrent medical conditions, bilobectomy, and pneumonectomy | 298 | 147 | 151 | 65.0 years (IQR 57.0-73.0) | Never 177; ever 121 | NSCLC overall; detailed histologic subtype distribution not reported | Resectable pathologic stage I-IIIA; stage I-II 262, stage III 36 | Lobectomy, wedge resection, or segmentectomy | VATS or open thoracotomy; converted VATS classified as open thoracotomy | Albumin; NLR; PLR | Albumin measured directly; NLR = neutrophil count / lymphocyte count; PLR = platelet count / lymphocyte count | Peripheral blood | Within 1 week before surgery | No significant ROC-derived cut-off for NLR or PLR for postoperative adverse events; only continuous biomarker values were analyzed for PPC/infection outcomes | PPC versus no PPC; multivariable logistic regression using continuous clinical and biomarker variables | Composite PPC outcome defined according to STS/ESTS criteria | Atelectasis, ARDS, emphysema, pneumonia, pleural effusion, pulmonary embolism, prolonged air leak ≥7 days, and re-intubation | Within 30 days after operation | 77 with PPCs (25.8%); component counts: prolonged air leakage 28, pneumonia 22, atelectasis 12, emphysema 10, ARDS 10, pleural effusion 9, re-intubation 2, pulmonary embolism 1 | Age; sex; smoking; mFI-5; pathologic stage; BMI; albumin; sarcopenia; surgical options; type of resection; treatment modality | Yes | Yes | Age ≥60 years; smoking history; mFI-5; pathologic stage; albumin ≤3.5 g/L; sarcopenia; surgical approach | Smoking; sarcopenia and other clinical variables in PPC model; target inflammation-based biomarkers were not retained as independent PPC predictors | Sarcopenia for PPCs: 2.418 | 1.162-3.425 | NR | NR | No | Yes | |
| Jiang et al. [8] | 2023 | China | Single-center retrospective cohort study | January 2019 to December 2020 | Lung Cancer Center, West China Hospital of Sichuan University; single center | Patients with NSCLC undergoing pulmonary resection | Included: pathologically confirmed NSCLC, no neoadjuvant therapy, and pulmonary resection; excluded: stage IV disease or palliative surgery, preoperative pneumonia or antibiotic/hormone use within 1 month, and autoimmune disease | 1486 | 748 | 738 | 58.2 ± 9.7 years | Yes 151; no 1335 | Adenocarcinoma 1010; squamous cell carcinoma 386; other 92 | Pathological stage I 1082; II 324; III 80 | Pulmonary resection | VATS or open thoracotomy | SII; NLR; PLR | SII = platelet × neutrophil / lymphocyte | Peripheral blood | 1 day before surgery | Cut-off 483 (Youden index 0.41) | POP versus no POP; multivariable logistic regression with continuous SII; ROC analysis | POP outcome | POP diagnosed using clinical, radiographic, laboratory, and microbiologic criteria; severity graded by Clavien–Dindo | Within 30 days after surgery | 142 POP / 1486 total (9.5%) | Sex; age; smoking; diabetes; hypertension; CHD; COPD; BMI; tumor stage; histology; FEV1; DLco; operative time; blood loss; surgical approach | Yes | Yes | BMI; SII; diabetes; FEV1 | OR | OR 1.000 | 1.000-1.001 | AUC = 0.751 | Sensitivity 68.3%; specificity 72.7%; cut-off 483 | Yes | Yes | |
| Lan et al. [9] | 2017 | China | Single-center prospective observational cohort study | December 2014 to February 2016 | West China Hospital of Sichuan University; single institution | Patients with NSCLC undergoing radical lung cancer surgery | 435 patients were screened; 186 were enrolled; 174 pathologically confirmed NSCLC cases were included in the final analysis | 174 | 122 | 52 | 59.02 ± 11.33 years | Never 64; smoker 110 | NSCLC overall; adenocarcinoma 64, squamous cell carcinoma 96, others 14 | Pathological stage I 29, II 34, III 88, IV 23 | Radical lung cancer surgery, including sublobectomy, lobectomy, bilobectomy, pneumonectomy, and sleeve lobectomy | Thoracotomy | PLR; NLR | PLR = platelet / lymphocyte ratio; NLR = neutrophil / lymphocyte ratio | Peripheral blood / preoperative complete blood count | Preoperative | PLR cut-off 148.6; NLR cut-off 2.9 | High versus low PLR; high versus low NLR | Postoperative pulmonary complication framework | Pneumonia, bronchopleural fistula, respiratory failure, ARDS/ALI, atelectasis, any pulmonary complications; prolonged air leak also reported separately | Early postoperative in-hospital period; 1-year follow-up was used for survival outcomes only | Any pulmonary complications: PLR low 30/103 vs PLR high 35/71; NLR low 30/105 vs NLR high 35/69; pneumonia: PLR low 22/103 vs PLR high 29/71; NLR low 25/105 vs NLR high 26/69 | Age; sex; BMI; smoking; ASA; lung function; comorbidities; clinical symptoms; adjuvant therapy; pathological type; pathological stage; type of resection; sleeve lobectomy; tumor location | Yes | No for PPC multivariable model; Yes for survival Cox models | Not applicable for PPC effect estimates; Cox models were adjusted for survival outcomes rather than PPC outcomes | Raw frequency comparison for PPCs; HR for OS/RFS | Not directly reported for PPC as OR/RR/HR | Not applicable for PPC | PLR AUC 0.628; NLR AUC 0.648 | PLR cut-off 148.6 and NLR cut-off 2.9 were derived from ROC analyses for survival prediction | No | Yes | |
| Miao et al. [10] | 2024 | China | Single-center retrospective cohort study | March 2021 to March 2022 | Affiliated Hospital of Nantong University; single center | Adults undergoing elective lung resection surgery | Included: age ≥18 years, elective VATS lung resection, general anesthesia with double-lumen tube, extubation in the operating room, ASA I-III; excluded: respiratory infection within 3 months, wedge resection, missing SII-related laboratory data, and hepatic disease affecting prealbumin | 386 | 156 | 230 | 62.0 years (IQR 55.0-68.0) | Reported | Not reported | Not reported | Segmentectomy or lobectomy | Video-assisted thoracoscopic surgery | SII; prealbumin | SII = platelet × neutrophil / lymphocyte; prealbumin measured directly | Peripheral blood | Preoperative | Prealbumin 227; SII 261; combined-model cut-off not directly extractable | POP versus no POP; multivariable logistic regression; combined SII-plus-prealbumin model | POP outcome | Postoperative pneumonia | Within 7 days after surgery | 57 with POP / 386 total | Age; sex; BMI; ASA; smoking; drinking; hypertension; diabetes mellitus; coronary heart disease; COPD; perioperative clinical/laboratory variables | Yes | Yes | SII; prealbumin; other significant clinical variables (full adjusted set not completely reported in the available text) | OR | SII 1.38; prealbumin 0.80 | 1.19-2.83; 0.37-0.89 | Prealbumin AUC 0.60; SII AUC 0.69; combined-model AUC 0.79 | Prealbumin cut-off 227, sensitivity 65.2%, specificity 54.1%; SII cut-off 261, sensitivity 78.3%, specificity 42.8%; combined-model performance superior, but the exact cut-off, sensitivity, and specificity were not directly extractable | Yes | No | |
| Mizuno et al. [6] | 2012 | Japan | Retrospective cohort study | July 2004 to March 2011 | Gifu University and Juntendo University School of Medicine; two centers | Patients with clinical IPF undergoing pulmonary resection for primary lung cancer | Among 1444 patients with primary lung cancer, 62 had clinical IPF; 52 patients who underwent pulmonary resection, excluding pneumonectomy and wedge resection, were analyzed | 52 | 50 | 2 | Not reported (group-wise values reported) | Smoking exposure reported as pack-years | Primary lung cancer histology NR | Primary lung cancer with clinical IPF | Segmentectomy, lobectomy, or bilobectomy | Thoracotomy with thoracoscopic assistance | CRP | CRP measured directly | Peripheral blood | Preoperative | No formal cut-off reported | PAE versus no PAE; multivariable logistic regression | Pulmonary complication framework focused on PAE of IPF | Postoperative acute exacerbation of IPF | Postoperative period | 7 patients with PAE / 52 total (13.5%) | Preoperative predictive factors and perioperative management variables, especially fluid management | Yes | Yes | Intraoperative fluid balance; preoperative CRP; perioperative variables | OR | CRP 1.280 | 95% CI not directly extractable from the abstract; p = 0.048 reported | AUC NR | NR | No | Yes | |
| Ruan et al. [11] | 2025 | China | Single-center retrospective cohort study | January 2019 to 2023 (month inconsistently reported in the abstract and Methods) | The First People’s Hospital of Jiande; single center | Patients with NSCLC undergoing lung resection | Included: NSCLC treated with lung resection; excluded: benign disease, repeat surgery, >1 surgery within 1 month, preoperative oncologic therapy, second primary lung tumors, infection/autoimmune disease requiring antibiotics or hormones, stage IV disease or palliative surgery, and transfer to another hospital | 568 overall; 252 after PSM | 157 after PSM | 95 after PSM | 63.89 ± 11.43 years after PSM | Reported | Histology reported | Re-staged according to the 8th edition of the TNM classification | Lung resection | VATS approach reported | Neutrophil count; lymphocyte count; monocyte count; platelet count; NLR; PLR; LMR; SII | SII = platelet × neutrophil / lymphocyte; PLR and LMR calculated using standard formulas | Peripheral blood | Within 3 days before surgery | PLR 140.315; LMR 4.875 | POP versus no POP after 1:3 propensity score matching; multivariable logistic regression; ROC analysis | POP outcome | Postoperative pneumonia; 1-month chest CT-based radiographic outcomes also reported | Postoperative period for POP; 1 month for chest CT outcomes | 72 POP / 568 overall; after PSM 66 POP vs 186 non-POP | Sex; age; BMI; smoking history; comorbidities; TNM stage; resection site; resection type; lymph node variables; operative variables; inflammatory biomarkers | Yes | Yes | PLR; LMR; preoperative chest CT findings; other significant variables (final adjusted set not completely reported in the available text) | OR | PLR 0.988; LMR 0.618 | 0.980-0.996; 0.494-0.772 | PLR AUC 0.780; LMR AUC 0.729 | PLR cut-off 140.315, sensitivity 66.7%, specificity 88.2%; LMR cut-off 4.875, sensitivity 56.1%, specificity 80.1% | Yes | No | |
| Shen et al. [12] | 2017 | China | Single-center retrospective cohort study | January 2014 to December 2015 | Department of Thoracic Surgery, single center | Patients with NSCLC undergoing lung cancer lobectomy | Excluded: advanced-stage or unknown-stage cancer, secondary lung cancer, non-NSCLC histology, and incomplete or missing data | 1058 | 587 | 471 | 61.3 ± 10.0 years | Current smokers 225 | NSCLC overall; adenocarcinoma 748, squamous cell carcinoma 273, other 37 | Non-advanced, known-stage NSCLC after exclusions; the exact stage distribution was not directly extractable from the available baseline summary | Lobectomy | VATS or open surgery | NLR; WBC; A/G ratio; platelet | NLR = neutrophil percentage / lymphocyte percentage within the total white blood cell count | Peripheral blood | Within 3 days before surgery | No formal cut-off reported | POP versus no POP; binary logistic regression for POP and ordinal logistic regression for POP severity | POP outcome | POP; severity graded using the Clavien–Dindo classification | Within 30 days after surgery | 150 POP / 1058 total (14.2%) | Age; sex; smoking history; ppoFEV1%; ppoDlco%; ASA class; CCI; BMI; stage; pathology; WBC; NLR; A/G ratio; platelet; surgical approach; blood loss; operative time | Yes | Yes | Operation time; blood loss; CCI >3; ASA >3; ppoDlco%; ppoFEV1%; WBC; NLR; A/G ratio; platelet; surgical approach; COPD; cardiovascular disease; diabetes; early stage; sex; age | OR | NLR 2.171 | 1.721-2.737 | NR | NR | Yes | Yes | |
| Abbreviations: A/G, albumin/globulin ratio; ALI, acute lung injury; ARDS, acute respiratory distress syndrome; ASA, American Society of Anesthesiologists; AUC, area under the curve; BMI, body mass index; CBC, complete blood count; CCI, Charlson comorbidity index; CHD, coronary heart disease; CI, confidence interval; COPD, chronic obstructive pulmonary disease; CRP, C-reactive protein; CT, computed tomography; DLco, diffusing capacity of the lungs for carbon monoxide; ECMO, extracorporeal membrane oxygenation; FEV1, forced expiratory volume in 1 second; HR, hazard ratio; ICU, intensive care unit; IPF, idiopathic pulmonary fibrosis; IQR, interquartile range; LMR, lymphocyte-to-monocyte ratio; NLR, neutrophil-to-lymphocyte ratio; NR, not reported; OLV, one-lung ventilation; OR, odds ratio; OT, open thoracotomy; PAE, postoperative acute exacerbation; PLR, platelet-to-lymphocyte ratio; POP, postoperative pneumonia; PPCs, postoperative pulmonary complications; ppoDlco%, postoperative predicted diffusion capacity for carbon monoxide of the lung; ppoFEV1%, postoperative predicted forced expiratory volume in 1 second; PSM, propensity score matching; ROC, receiver operating characteristic; SD, standard deviation; SII, systemic immune-inflammation index; TNM, tumor-node-metastasis; VATS, video-assisted thoracoscopic surgery; WBC, white blood cell.  **Footnotes:** For Chang et al. [7], adjusted PPC effect estimates were not directly available for the target inflammation-based biomarkers. For Lan et al. [9], the reported cut-off values and AUCs were derived for survival prediction rather than PPC prediction. For Miao et al. [10], the exact combined-model cut-off and full performance details were not clearly reported in the article. For Ruan et al. [11], the study period was reported inconsistently between the abstract and the Methods; this discrepancy is noted in the table. NR indicates not reported in the original article. | | | | | | | | | | | | | | | | | | | | | | | | | | | | | | | | | | | | | |  |
